# Discordant CSF/plasma HIV-1 RNA in individuals on virologically suppressive antiretroviral therapy in Western India

**DOI:** 10.1097/MD.0000000000009969

**Published:** 2018-02-23

**Authors:** Ameet N. Dravid, Kartik Natrajan, Milind M. Kulkarni, Chinmay K. Saraf, Uma S. Mahajan, Sachin D. Kore, Niranjan M. Rathod, Umakant S. Mahajan, Rustom S. Wadia

**Affiliations:** a Department of Medicine, Ruby Hall Clinic; b Department of Medicine, Poona Hospital; c Department of Medicine, Noble Hospital; d Precision Diagnostics and Biosciences, Pune; e Department of Dermatology, Ashwini Sahakari Rugnalaya, Solapur; f Department of Medicine, Apex Hospital, Kolhapur; g GeneOmbio Technologies Private Limited, Pune, Maharashtra, India.

**Keywords:** antiretroviral therapy (ART), CNS penetration effectiveness (CPE) score, CSF/plasma HIV-1 RNA discordance, HIV encephalopathy, low-level viremia (LLV), nadir CD4 count, protease inhibitors

## Abstract

Aim of this study was to estimate the prevalence of cerebrospinal fluid (CSF)/Plasma HIV-1 RNA discordance in virologically suppressed individuals presenting with incident neurologic symptoms.

In this retrospective cohort study conducted between March 1, 2009, and March 1, 2017, HIV-1 infected adults exposed to atleast 12 months of antiretroviral therapy (ART) and having plasma viral load (VL) <1000 copies/mL (virologically suppressed) were included. Among these, individuals presenting with neurologic symptoms during follow-up were assessed for CSF/Plasma HIV-1 RNA discordance by measuring HIV-1 RNA in collected plasma and CSF samples. CSF/plasma HIV-1 RNA discordance was defined as either detectable CSF HIV-1 RNA (VL > 20 copies/mL) with an undetectable plasma RNA (complete viral suppression, VL ≤20 copies/mL) or CSF HIV-1 RNA ≥ 0.5 log_10_ higher than plasma RNA when plasma VL was between 20 and 1000 copies/mL (low-level viremia, LLV).

Out of 1584 virologically suppressed patients, 71 (4.4%) presented with incident neurologic symptoms. Twenty out of 71 (28.2%) patients were diagnosed with CSF/Plasma HIV-1 discordance. Median plasma and CSF VL in patients with discordance was 120 [interquartile range (IQR): <20 to 332.5] and 4250 (IQR: 2550.0– 9615.0) copies/mL, respectively. All 9 individuals in which CSF HIV-1 genotypic resistance testing was done showed mutations that would compromise efficacy of prescribed ART regimen. Prevalence of CSF/plasma HIV-1 RNA discordance was higher among neurologically symptomatic patients with plasma LLV as compared with those with complete viral suppression (70% vs 11.8%, *P* < .001). The risk of discordance was also greater in patients who received protease inhibitor (PI) containing ART (*P* < .001) and those on ART regimens with central nervous system (CNS) penetration effectiveness (CPE) value <6 (*P* = .006).

CSF/plasma HIV-1 RNA discordance indicates replication of HIV-1 that has adapted to the CNS or has developed antiretroviral drug resistance. Larger studies should be performed to study incidence of discordance in India. This will help in managing patients presenting with neurologic symptoms on suppressive ART with appropriate neuroeffective therapy.

## 1 Introduction

The central nervous system (CNS) is a reservoir for HIV, which is established very early during the course of HIV infection.^[1,2]^ With the advent of potent antiretroviral therapy (ART), virologic suppression is achieved in plasma and cerebrospinal fluid (CSF)^[3,4]^ and the incidence of HIV-1 associated dementia has declined dramatically.^[5]^ However, milder forms of HIV associated neurocognitive disorders persist in 25% to 50% people with chronic HIV-1 infection.^[5]^ Episodes of severe neurologic impairment developing in patients on virologically suppressive ART have also been reported.^[6,7]^ One reason for this could be the limited distribution of ART drugs into CNS due to restriction at the blood–brain and blood–CSF barriers. If the concentrations of ART drugs in the CNS are subtherapeutic, HIV-1 could replicate at low levels, leading to viral neurotoxicity, chronic sustained immune activation, and evolution of drug-resistant CNS HIV.^[8–10]^ Persistent HIV-1 RNA in CSF with or without neurologic symptoms in spite of well controlled plasma HIV-1 RNA (CSF/Plasma HIV-1 RNA discordance) has been reported from Europe and North America.^[11–16]^ Independent evolution of drug resistant CNS HIV has also been described.^[17,18]^ However, there are sparse data on neurosymptomatic CSF/plasma HIV-1 discordance from countries outside these regions, such as India. The prevalence of CSF/plasma HIV-1 discordance is estimated at 12% to 21% among adults undergoing clinically indicated lumbar punctures (LPs).^[14,15]^ The objective of this study was to estimate the prevalence of CSF/Plasma HIV-1 RNA discordance in virologically suppressed Indian patients presenting with incident neurologic symptoms and associated risk factors.

## 2 Materials and methods

### 2.1 Study design

This was a retrospective cohort study conducted between March 1, 2009, and March 1, 2017, at 3 private, tertiary-level hospitals and research centers in Pune, Western India (Ruby Hall Clinic, Poona Hospital, and Noble Hospital).

### 2.2 Settings and patient characteristics

All 3 private hospitals provide clinical care, diagnostic, and treatment services. The patients are followed regularly on the basis of their clinical presentations and treatment duration. The demographic, clinical, and laboratory data were abstracted from the electronic records of the hospital database. The approval for data analysis was obtained from the Ethics Committees of all 3 hospitals. We retrospectively compiled data of all patients who had at least 12 months of stable ART and plasma HIV-1 viral load (VL) <1000 copies/mL, a level that is considered to be virologically suppressed as per WHO ART guidelines.^[19]^ Among these, individuals presenting with neurologic symptoms during follow-up such as imbalance during walking, memory and speech disturbance, intractable headache, pain and sensory disruption, seizures, partial or complete paralysis of limb, and loss of control over bladder and bowel were identified and their inpatient case files were studied. Laboratory and imaging data including routine biochemical investigations, CD4 count, CSF studies, and magnetic resonance imaging (MRI, GE 1.5 Tesla Signa model in 2 hospitals and Phillips 1.5 Tesla model in 1 hospital) were abstracted for analysis. Follow-up data of each patient were censored at the time of development of neurologic symptoms or loss of virologic suppression (plasma VL on ART > 1000 copies/mL) or loss to follow-up or completion of cohort duration or death of patient.

### 2.3 Diagnosis and clinical management

Criteria used to diagnose common neurologic conditions in our cohort were as follows:

1) CNS tuberculosis (TB): CSF automated liquid TB culture, CSF TB real-time polymerase chain reaction (PCR) assay (Xpert MTB/RIF, Cepheid, USA), and MR imaging.
2) Cerebral toxoplasmosis and progressive multifocal leucoencephalopathy (PML): MR imaging, stereotactic brain biopsy, and CSF JC virus DNA in case of PML.
3) Cryptococcal meningoencephalitis: CSF Cryptococcal antigen and CSF fungal culture.
4) HIV encephalopathy (HIV-E): MR imaging and the absence of other diagnoses.
5) Viral encephalitis such as herpes simplex virus (HSV) and Varicella zoster virus (VZV): MR imaging and CSF PCR for viral DNA.
6) Neurosyphilis: CSF WBC count of 20 cells/μL or greater and a reactive CSF venereal disease research laboratory test.
7) Cerebrovascular diseases: MR imaging.

In patients admitted with neurologic symptoms, paired plasma and CSF samples were collected for HIV-1 RNA estimation as a standard of care at all 3 hospitals. HIV RNA was measured by real-time PCR [NucliSENS Easy Q real-time nucleic acid sequence based amplification (NASBA); BioMérieux, France, detection limit -20 to 10,000,000 copies/ml]. Patients presenting with neurologic symptoms were further classified into those with complete plasma viral suppression (PVL <20 copies/mL) and those with low-level viremia (LLV; PVL: 20–1000 copies/mL) as per their latest plasma VL values. CSF/Plasma HIV-1 RNA discordance was defined as either detectable CSF HIV RNA (CSF VL > 20 copies/mL) with an undetectable plasma RNA (plasma VL ≤20 copies/mL) or CSF HIV RNA ≥ 0.5 log_10_ higher than plasma RNA when plasma VL was between 20 and 1000 copies/mL.^[14]^ In a subset of patients with discordance, CSF HIV-1 genotypic resistance testing was performed. Protease, reverse transcriptase (RT), and integrase genes were sequenced using an automated population-based full sequence analyser (ABI 3130 Genetic Analyser; PE Applied Biosystems, minimum detection limit for successful sequencing - 1000 copies/mL). Resistance testing was not attempted in the plasma virus in view of low VL and cost constraints. Effectiveness of ART in CNS was determined by applying the revised CNS penetration effectiveness score 2010 to current suppressive ART regimen.^[20]^ CPE score was calculated by assigning a predefined number of points to each component of ART regimen. Among neurologically symptomatic individuals, those showing CSF/Plasma HIV-1 RNA discordance were taken as cases and the rest were controls.

### 2.4 Statistical methods

Baseline characteristics for continuous variables were summarized using median and interquartile range (IQR), and for noncontinuous variables using frequency and percentages. Continuous variables were compared using a median test. Categorical variables were compared using Chi-square test and Fishers exact test. Incidence of neurologic disease and corresponding 95% confidence intervals (CIs) were estimated. All analyses were performed using STATA version 12.1 (Statacorp LLC, Texas).

## 3 Results

**Figure 1:**
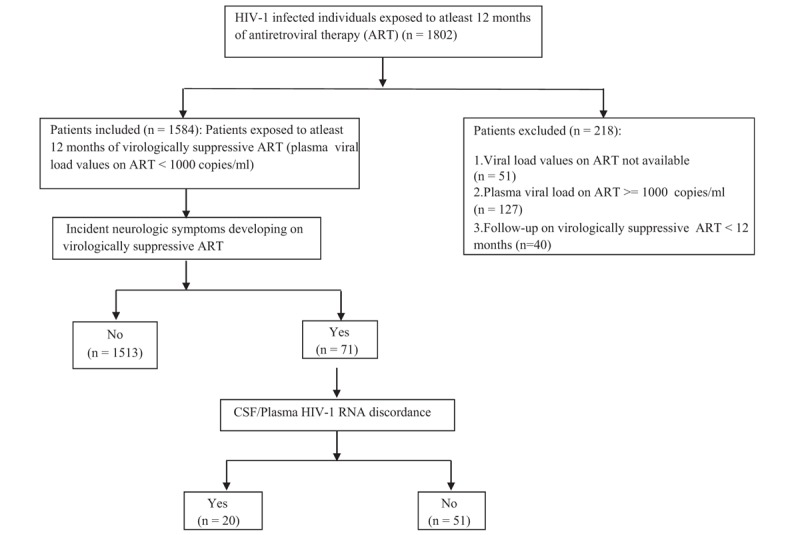
Flow chart depicting identification of individuals with neurosymptomatic CSF/Plasma HIV-1 RNA discordance in Pune cohort. CSF = cerebrospinal fluid.

One thousand five hundred eighty-four virologically suppressed HIV-1 infected adults (35.7% females) with 7139.17 person-years of follow-up on suppressive ART were included in the study (Fig. 1). Median age was 39 years (IQR = 33.0, 46.0), median pre-ART CD4 count was 174.5 (IQR = 83.0–257.0) cells/mm^3^, and median nadir CD4 count was 156 (IQR = 75.0–241.0) cells/mm^3^. About 31.9% patients had a history of TB before starting ART. A total of 133 neurologic conditions were reported in 107 (6.8%) patients before starting ART. The most commonly reported baseline neurologic conditions were CNS TB (42/107), HIV encephalopathy (24/107), Cryptococcal meningitis (20/107), CNS toxoplasmosis (21/107), and PML (6/107). Median duration of follow-up since HIV diagnosis was 62 (IQR = 36.0–90.0) months, median duration of ART was 56 (IQR = 32.0–84.0) months, and median duration of viral suppression was 50 (IQR = 26.0–74.0) months. Non-nucleoside reverse transcriptase inhibitor (NNRTI)-containing [2 nucleoside reverse transcriptase inhibitors (NRTIs) and 1 NNRTI] and protease inhibitor (PI)-containing regimens (2 NRTIs and 1 ritonavir-boosted (PI) or 1 boosted PI with integrase inhibitor) were prescribed to 1218 (76.9%) and 360 (22.7%) patients, respectively. Tenofovir with either Emtricitabine or Lamivudine was the most commonly used NRTI backbone. Efavirenz was the most common NNRTI and ritonavir-boosted Atazanavir (ATV/r) the most common PI used in our cohort. One thousand four hundred forty of 1584 (90.9%) patients had complete viral suppression, while 144 of 1584 (9.1%) had LLV at the time of latest plasma VL estimation. Seventy-eight of 1218 (6.4%) and 66 of 360 (18.3%) patients on NNRTI and PI-based therapy had LLV, respectively. Median CPE score of ART regimen was 6 (IQR = 6.0–7.0). Two percent patients were on ART regimens with CPE <6.

### 3.1 Incident neurological symptoms

**Figure 2:**
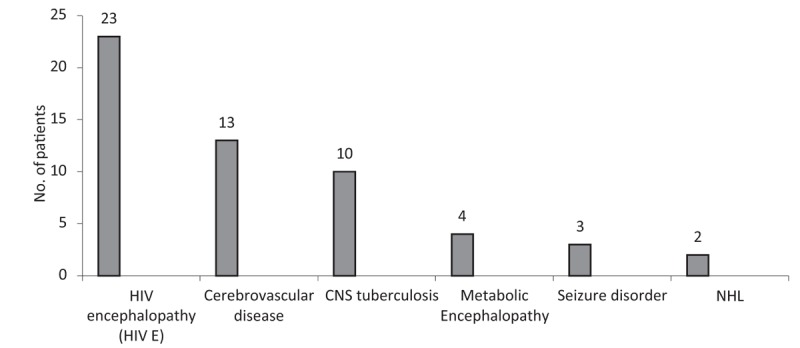
Incident neurologic disease (n = 55). Cerebrovascular disease includes clinical conditions such as ischemic stroke, hemorrhagic stroke, and intracranial bleed. Metabolic encephalopathy includes clinical conditions such as hepatic encephalopathy, uremic encephalopathy, septic encephalopathy, and hyponatremic encephalopathy. X axis – Neurologic diagnosis. Y axis – Total number of patients. HIV-E = HIV encephalopathy, NHL = non-Hodgkin lymphoma.

Of the 1584 virologically suppressed individuals, 71 (4.4%) patients had neurologic symptoms during follow-up and required inpatient care (Fig. 1). Fifty-five of 71 (77.5%) patients were diagnosed to have an incident neurologic disease [7.71 (95% CI): 5.920–10.043] episodes per 1000 person-years]. HIV encephalopathy (HIV-E), CNS TB, and cerebrovascular disease (ischemic stroke or intracranial hemorrhage) were the most common incident neurologic diseases (Fig. 2). On basis of clinical findings, biochemical investigations, neuroimaging, and CSF studies, there was no significant neurologic diagnosis in 16 patients. About 71.8% patients with incident neurologic symptoms had complete viral suppression, while 28.2% had LLV.

### 3.2 CSF/plasma HIV-1 RNA discordance

Twenty of 71 (28.2%) patients with neurologic symptoms had CSF/Plasma HIV-1 RNA discordance (12 men and 8 women). Demographic data of patients with and without discordance have been elucidated in Table 1. Imbalance during walking, forgetfulness, and tremor of hands were the most common presenting symptoms of patients presenting with discordance. Twelve of 20 (60%) had subacute onset of symptoms. Eight of 20 (40%) patients presented with acute symptoms such as seizures or altered level of consciousness. Median duration of follow-up since HIV diagnosis in patients with discordance was 74 (IQR: 37.0–109.0) months, median duration of ART was 73 (IQR: 31.0–108.0) months, and median duration of viral suppression was 25 (IQR: 19.0–38.0) months. Except for 1 case, all individuals with CSF/plasma HIV discordance were on PI-containing ART. Median nadir CD4 count and median CD4 count at the time of discordance was 54.5 (IQR: 29.0–102.3) cells/mm^3^ and 352 (IQR: 200.3–505.8) cells/mm^3^, respectively. Median plasma and CSF VL in patients with CSF/plasma HIV discordance was 120 (IQR: <20 to 332.5) and 4250 (IQR: 2550.0–9615.0) copies/mL, respectively (Table 2). Abnormal CSF studies (high CSF protein, abnormal CSF glucose, or lymphocytic pleocytosis) were found in 18 of 20 (95.0%) patients. Median CSF protein was 97.5 mg/dL (IQR: 80.25–107.75), median CSF sugar was 54 mg/dL (IQR: 45.75–56.50), and median CSF cell count was 18 cells/mm^3^ (IQR: 10.0–35.0). Most common MRI findings in patients with CSF/plasma HIV discordance were generalized cerebral atrophy and asymmetrical, nonenhancing periventricular white matter hyperintensities on T2 and FLAIR images. Three patients had HIV-related meningoencephalitis on MRI. CSF samples from 11 of 20 (55%) patients underwent sequencing for the detection of HIV-1 resistance-associated mutations (RAMs). Two samples could not be amplified. All 9 samples tested showed RAMs that reduced efficacy of 1 or more antiretroviral drugs from current ART regimen. The most common CSF mutation in RT gene was M184 V/I, which was seen in all 9 samples. Thymidine analog mutations (TAMs) were present in 7 cases (77.78%), most commonly T215Y/V/F, K219E/Q, or D67N/T (Table 2). The most common NNRTI mutations were Y181C and K103N/S, which were present in 5 samples each. The most common major PI mutations were V82A and I50L, which were present in 2 patients each.^[21,22]^ One sample showed presence of N155H mutation in the integrase gene.^[22]^ Triple class resistance (resistance to NRTI, NNRTI, and PI drugs) was seen in 5 (55.6%) CSF samples (patient number 5, 6, 10, 11, and 16, Table 2). In the remaining 4 samples (patient number 3, 12, 17, and 20, Table 2), NRTI and NNRTI resistance was seen in CNS virus. All cases of discordance were seen in patients presenting with HIV-E. There was no case of secondary CSF escape, defined as discordant CSF and plasma HIV-1 RNA levels in the context of a new infection, in our cohort.

**Table 1:**
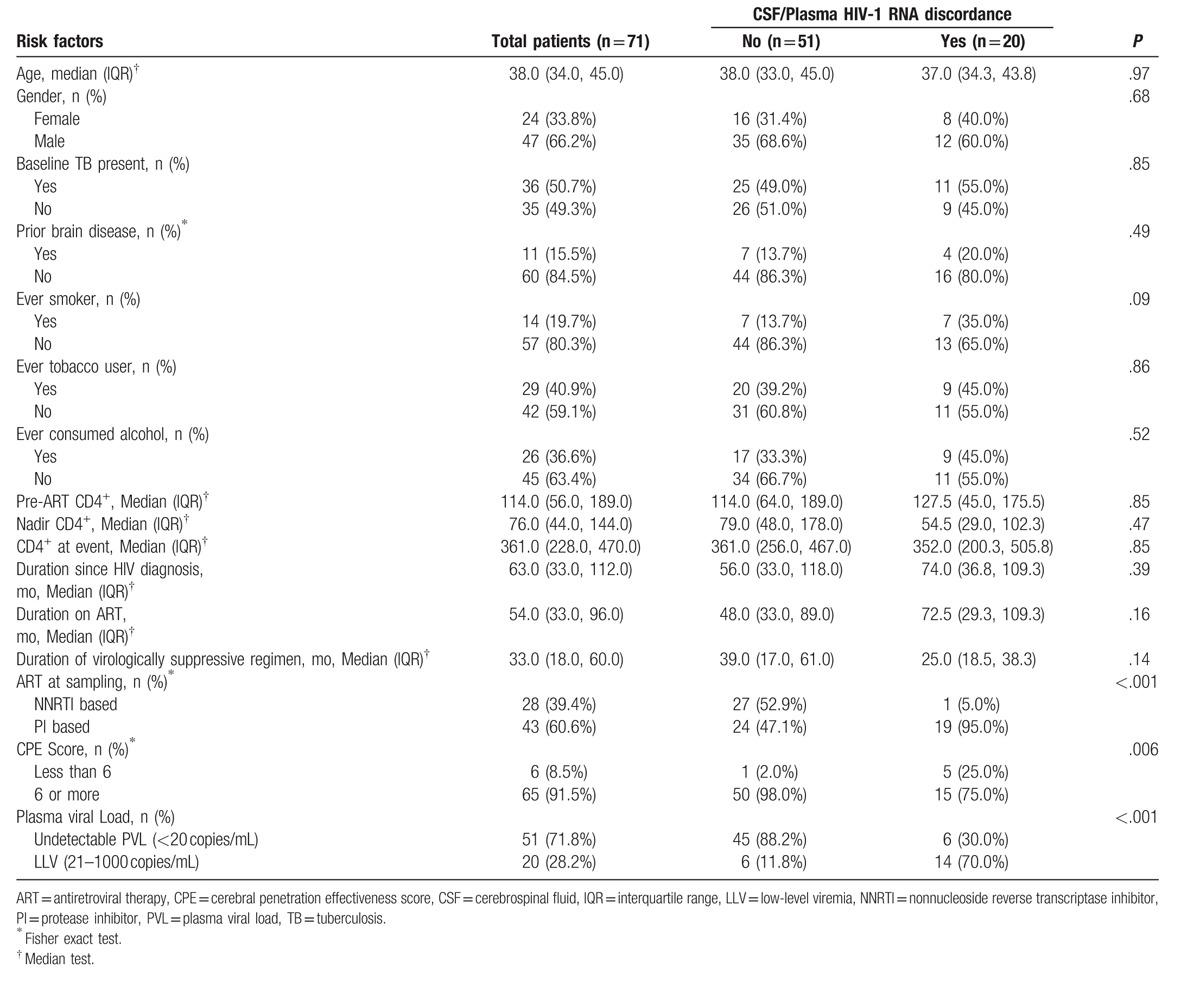
Factors associated with neurosymptomatic CSF/plasma HIV-1 RNA discordance.

**Table 2:**
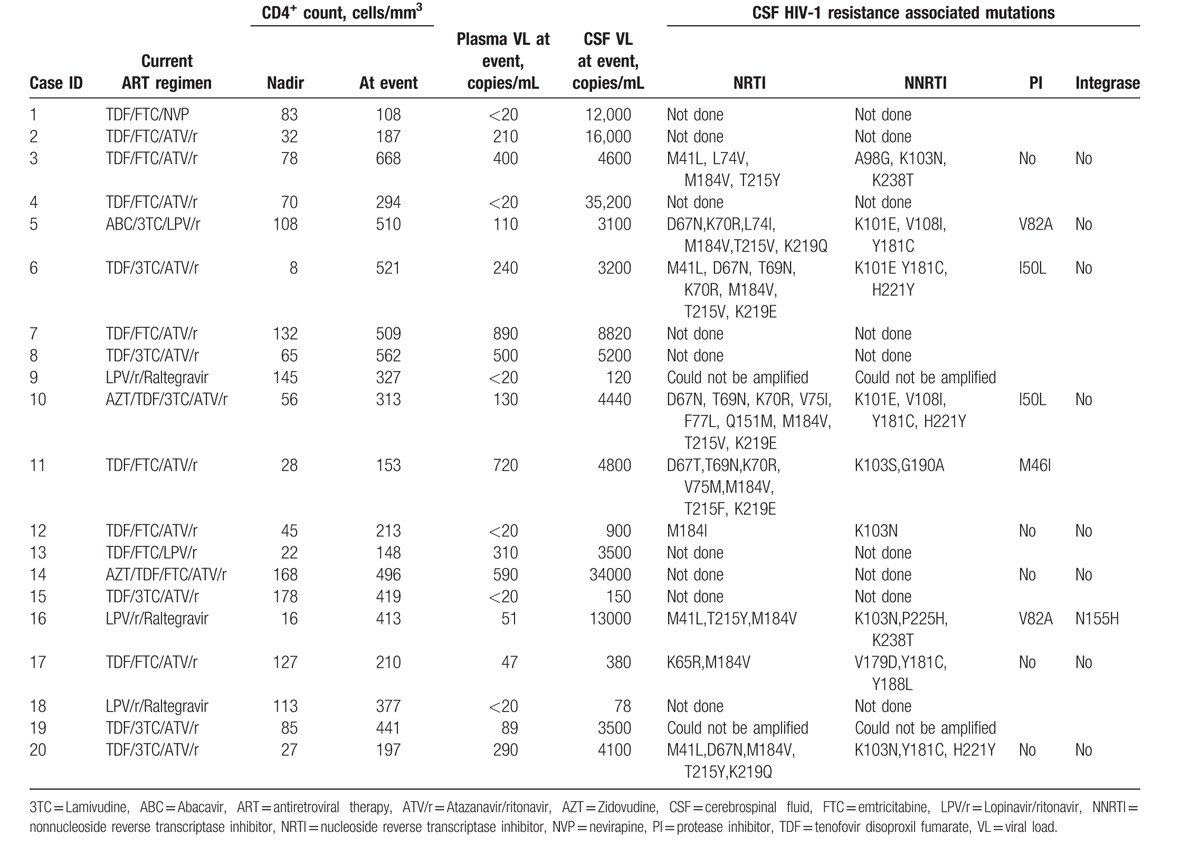
CD4^+^-T-cell counts, plasma, and CSF HIV-1 viral loads in individuals with CSF/Plasma HIV-1 RNA discordance.

### 3.3 Factors associated with neurosymptomatic CSF plasma HIV-1 RNA discordance

Results presented in Table 1 summarize that neurosymptomatic CSF/plasma HIV-1 RNA discordance was not associated with age, gender, presence of baseline TB, baseline brain disease, nadir CD4 count, and duration of ART regimen. Prevalence of CSF/plasma HIV-1 RNA discordance was higher among neurologically symptomatic patients with plasma LLV than those with complete viral suppression (70% vs 11.8%, *P* < .001). The risk of neurosymptomatic CSF/plasma HIV-1 discordance was greater in patients who received PI-containing ART (*P* < .001). Individuals who took ART regimens with CPE values <6 were more likely to develop discordance (*P* = .006).

## 4 Discussion

This study reports prevalence of neurosymptomatic CSF/Plasma HIV-1 RNA discordance in virologically suppressed adults in western India. Incident neurologic deterioration requiring hospitalization was an uncommon but significant event in our patients on virologically suppressive ART. Of the 71 patients who developed neurologic symptoms during the period of observation, 20 (28.2%) had CSF/Plasma HIV-1 RNA discordance.

### 4.1 CSF/plasma HIV-1 RNA discordance

This entity also known as CSF HIV escape was first described by Canestri et al,^[11]^ followed by a second case series by Peluso et al.^[13]^ Recently, cases of CSF/Plasma HIV-1 discordance have been described by Nightingale et al^[15]^ and Mukerji et al^[16]^ In addition, there have been multiple individual case reports^[6,7,23–29]^ of CSF/Plasma HIV-1 discordance. To the best of our knowledge, our study is one of the first reports of a large number of neurosymptomatic CSF/plasma HIV-1 discordance cases from a resource-limited setting. As compared to the reported case series,^[11,13]^ we have described episodes of discordance as a part of incident neurologic disease, developing in patients on suppressive ART. Published case reports of symptomatic CSF/plasma HIV discordance described patients taking PI monotherapy or ART regimens, which are no longer recommended.^[11,27–29]^ In our series, all cases of CSF/plasma HIV-1 discordance occurred among patients taking WHO-recommended triple-drug NNRTI or PI-containing regimens.^[19]^ In our case series, we have studied CSF/plasma HIV-1 discordance in patients with plasma VL <1000 copies/mL (LLV or complete viral suppression), while other studies have included patients with complete viral suppression, plasma LLV, and high-level viremia (plasma VL >1000 copies/mL).^[15,16]^

Mechanisms of CSF/plasma HIV discordance remain poorly defined. One hypothesis suggests that discordance can be seen in individuals who fail to show durable viral suppression on ART. In such individuals, drug-resistant plasma HIV secondarily infects the CNS and subsequent replication of CNS HIV leads to discordance.^[30]^ Another hypothesis is that more advanced immune suppression (as estimated by a low nadir CD4^+^ T-cell count) eases HIV entry into the CNS. In the CNS, HIV productively replicates within perivascular macrophages and microglia (M tropic HIV). The administration of ART drugs that reach higher concentrations in the CNS better suppress viral replication in these cells, although integrated provirus persists. Suboptimal adherence to ART or switch to drugs with lower distribution into the CNS allows the neurologic reservoir of HIV to replicate at low levels. Suboptimal CNS penetration of ART and multidrug-resistant CNS HIV leads to compartmentalized infection, which manifests in its severest form as HIV encephalopathy.^[30,31]^ However, in our study, we did not find an association between nadir CD4 count and neurosymptomatic CSF/plasma HIV-1 discordance (*P* = .47).

### 4.2 Presence of plasma LLV and CSF/Plasma HIV-1 RNA discordance

Our results showed a higher prevalence of CSF/plasma HIV-1 RNA discordance among neurologically symptomatic patients with plasma LLV as compared with those with complete viral suppression (70% vs 11.8%, *P* < .001). This finding was also seen in a study conducted by Nightingale et al^[15]^ and could signify suboptimal suppression in both plasma and CSF. Intermittent or persistent plasma LLV occurs in up to a quarter of ART-treated patients.^[32]^ Persistent plasma LLV between 50 and 999 copies/mL is associated with an increased risk of virologic failure.^[33]^ Accumulation of resistance mutations during LLV^[34]^ may increase risk of neurovirulence. Definition of virologic suppression used for patients in our cohort^[19]^ is different from that used in resource rich settings^[35]^ (plasma VL < 1000 copies/mL as compared with plasma VL < 200 copies/mL). This leads to a higher prevalence of plasma LLV in our cohort, especially among patients on PI-based therapy (18.3% vs 6.4% in patients on NNRTI-based therapy, *P* < .001). Plasma LLV does not warrant optimization of ART in India as it does in resource-rich settings. This could be the reason for higher prevalence of CSF/Plasma HIV-1 RNA discordance seen in our cohort compared with earlier studies (28.2% vs 12–21%).^[14,15]^ The treatment options for individuals with plasma LLV and neurosymptomatic CSF/plasma HIV-1 discordance must include strict adherence to ART to prevent plasma and CSF blips, complete suppression of plasma VL by changing ART regimen as per resistance testing report, and use of regimens with better CNS penetrability (CPE score of ≥6), in order to prevent loss of neurocognitive functions.

### 4.3 ART regimen and CSF/Plasma HIV-1 RNA discordance

Use of PI-based ART was associated with increased prevalence of neurosymptomatic CSF/Plasma HIV-1 discordance. In the present study, except for 1 case, all individuals with CSF/plasma HIV-1 discordance were on PI-containing ART. Out of 360 individuals who were on PI-based ART in our cohort, 19 (5.27%) developed neurosymptomatic CSF/Plasma HIV-1 discordance. Out of 43 patients who developed incident neurologic symptoms on PI-based ART, 19 (44.2%) had CSF/Plasma HIV-1 discordance (Table 1). As per the ART guidelines of WHO and Indian National AIDS Control Organization (NACO),^[36]^ PI-containing regimens (2 NRTI and 1 boosted PI or 1 boosted PI with 1 integrase inhibitor) are recommended as second-line treatment of HIV infected adults when NNRTI regimens (2NRTIs and 1 NNRTI) fail to suppress plasma VL. ATV/r is the preferred PI used in India, while boosted darunavir is preferred in resource-rich settings.^[35]^ ATV/r has subtherapeutic concentrations in CSF in a substantial proportion of adults and, accordingly, a low CPE value.^[37]^ The combination of CNS-compartmentalized HIV that may contain drug-resistant HIV archived during failed first-line therapy and subtherapeutic concentrations of ATV/r or another PI during second-line therapy could lead to functional dual or monotherapy. Among 9 patients undergoing CSF genotypic resistance testing in our cohort, 5 samples had triple-class resistance effectively leading to no active drug for viral suppression in the CNS compartment. In remaining 4 samples, NRTI and NNRTI resistance led to functional PI monotherapy in CNS compartment. Studies have shown a higher CSF HIV replication in patients taking double-boosted PI regimens, supporting possible functional monotherapy in the CNS.^[38]^ As PIs are substrates for drug efflux transporters that are expressed on brain microvascular endothelial cells and ependymal cells of choroid plexus, their concentrations in CNS can be subtherapeutic.^[39]^ These results supports the inference that shifting to a PI such as darunavir that has better CNS penetration than atazanavir^[40]^ and superior efficacy against triple-class resistant virus than lopinavir^[41]^ may reduce the risk of CSF/plasma HIV-1 RNA discordance.

### 4.4 CPE score and CSF/Plasma HIV-1 RNA discordance

The CPE method attempts to estimate the efficacy of ART drugs in the CNS. In cohort studies, higher CPE values, indicating better estimated efficacy of an ART regimen in the CNS, correlate with lower CSF HIV RNA.^[42]^ Evidence linking use of neuroactive ART regimens (regimens with higher CPE score) with improvement in cognitive performance,^[43–45]^ lower incidence of CSF/Plasma HIV-1 discordance,^[14,15]^ and prevention of multidrug-resistant CNS HIV^[46,47]^ has been mixed. However, our results show that using ART regimens with better CPE values (≥6) were beneficial, being associated with a lower prevalence of neurosymptomatic CSF/plasma HIV-1 discordance. As per the Mind Exchange Consensus Report, patients presenting with worsening cognitive impairment and detectable CSF HIV should consider modifying their ART regimen as per the CPE method provided other risk factors (e.g., poor adherence to medication, virologic drug resistance, and comorbidities) have been addressed.^[48]^ An “adjusted” CPE score has been proposed as a more relevant score in CSF/plasma HIV-1 discordance, as it takes into account resistance profiles for the calculation of CNS ART effectiveness.^[13]^ All 9 cases with genotyping from CSF isolates had mutations that would result in resistance to at least 1 prescribed ART drug, resulting in a lower “adjusted” CPE score.

Our study has several limitations. First, as for all retrospective studies, some episodes of incident neurologic disease may be unreported leading to measurement bias and underestimation of prevalence of CSF discordance. Second, milder neurologic symptoms such as headache may not have triggered LP and measurement of CSF HIV-1 RNA, leading to unaccounted cases of mildly symptomatic CSF discordance. Third, neuropsychological testing for cognitive impairment was not performed at baseline or follow-up in our cohort and hence milder forms of HIV-associated neurocognitive disease^[49]^ could not be identified and CSF discordance in these patients could not be studied. Screening for functional impairment^[48]^ and mood disorders^[50]^ was not performed as well. Fourth, patients were classified into LLV and complete viral suppression on the basis of latest plasma VL record. Longitudinal analysis of VLs to identify patients with intermittent LLV, persistent LLV, and durable suppression was not done. Some patients with intermittent LLV could have been classified as complete viral suppression as a result. Fifth, genotypic HIV-1 resistance testing of CNS virus was not performed for all patients with CSF/plasma HIV-1 discordance. In spite of these limitations, our cases of neurosymptomatic CSF/plasma HIV-1 RNA discordance clearly demonstrate that HIV persistence in the brain remains a concern among patients on virologically suppressive ART in India.

## 5 Conclusion

CSF/plasma HIV-1 RNA discordance remains understudied in low-income settings such as India despite a substantial HIV burden. Identification of these cases in our cohort will help bridge that gap and add to the growing body of literature on this uncommon but increasingly significant topic. These cases clearly illustrate that physicians in resource-limited settings such as India should perform CSF HIV-1 VL analysis in patients who develop neurologic symptoms while on PI-based ART with well-controlled plasma HIV. Association of CSF/plasma HIV-1 discordance with use of PI-containing ART and ART regimens with CPE score <6 should prompt use of newer PIs such as ritonavir-boosted darunavir and integrase inhibitors that have limited availability in resource-limited settings such as India. Higher prevalence of CSF/plasma HIV-1 RNA discordance among neurologically symptomatic patients with plasma LLV further strengthens the case for maintaining complete viral suppression on ART.
